# An Overview of the Cardioprotective Effects of Novel Antidiabetic Classes: Focus on Inflammation, Oxidative Stress, and Fibrosis

**DOI:** 10.3390/ijms24097789

**Published:** 2023-04-24

**Authors:** Dora Bianka Balogh, Laszlo Jozsef Wagner, Andrea Fekete

**Affiliations:** 1 Pediatric Center, MTA Center of Excellence, Semmelweis University, 1085 Budapest, Hungary; 2 MTA-SE Lendület “Momentum” Diabetes Research Group, 1083 Budapest, Hungary; 3 Department of Surgery, Transplantation, and Gastroenterology, Semmelweis University, 1085 Budapest, Hungary

**Keywords:** diabetes, cardioprotection, SGLT2 inhibitor, GLP-1 receptor agonist, DPP-4 inhibitor, inflammation, oxidative stress, fibrosis

## Abstract

Metabolic diseases, particularly diabetes mellitus (DM), are significant global public health concerns. Despite the widespread use of standard-of-care therapies, cardiovascular disease (CVD) remains the leading cause of death among diabetic patients. Early and evidence-based interventions to reduce CVD are urgently needed. Large clinical trials have recently shown that sodium-glucose cotransporter-2 inhibitors (SGLT2i) and glucagon-like peptide-1 receptor agonists (GLP-1RA) ameliorate adverse cardiorenal outcomes in patients with type 2 DM. These quite unexpected positive results represent a paradigm shift in type 2 DM management, from the sole importance of glycemic control to the simultaneous improvement of cardiovascular outcomes. Moreover, SGLT2i is also found to be cardio- and nephroprotective in non-diabetic patients. Several mechanisms, which may be potentially independent or at least separate from the reduction in blood glucose levels, have already been identified behind the beneficial effect of these drugs. However, there is still much to be understood regarding the exact pathomechanisms. This review provides an overview of the current literature and sheds light on the modes of action of novel antidiabetic drugs, focusing on inflammation, oxidative stress, and fibrosis.

## 1. Introduction

Metabolic diseases, including diabetes mellitus (DM), have reached alarming levels worldwide. The number of people living with DM has surpassed half a billion and is expected to rise to 700 million by 2045, in parallel with the obesity epidemic [1]. Diabetic patients have a more than doubled risk of developing cardiovascular disease (CVD) compared with the non-diabetic population [2]. The coexistence of type 2 DM (T2DM) and hypertension has long been established, with 85% of T2DM patients having increased blood pressure, predisposing them to cardiovascular complications [3]. The presence of diabetic kidney disease (DKD) further increases the incidence of CVD events and has become an independent risk factor for CVD [4], leading to the vicious circle of cardiorenal syndrome. Despite current standard-of-care therapies, CVD remains the leading cause of death, disability, and healthcare resource use among people with DM [5,6]. Early and evidence-based interventions aimed at reducing CVD are much needed.

Beyond lifestyle changes, tight glycemic control, normalization of hypertension, statins, and the use of renin–angiotensin–aldosterone system blockers have been the cornerstone in the treatment of DM in the last decades. Despite these gold-standard therapies reducing microvascular complications, the risk of CVD remains high, even in optimally managed patients [7,8]. Moreover, some oral antidiabetics have been associated with an increased cardiovascular risk, which prompted the U.S. Food and Drug Administration (FDA) to issue a guidance for industry in 2008, mandating requirements for demonstrating cardiovascular safety for all new anti-hyperglycemic medications in T2DM [9]. This boosted cardiovascular safety trials of novel compounds. In March 2020, the FDA released a draft guidance recommending a more comprehensive approach to evaluate the safety of new glucose-lowering drugs, that goes beyond cardiovascular risk. Consequently, the diabetes mellitus December 2008 guidance was withdrawn and replaced with this draft guidance [10].

In the past decade, there has been a real revolution in the field of oral antidiabetics, with various new compounds and novel mechanisms of action. The most promising and effective agents are sodium-glucose cotransporter-2 inhibitors (SGLT2i), glucagon-like peptide-1 receptors agonists (GLP-1RA), and dipeptidyl-peptidase-4 inhibitors (DPP-4i).

SGLT2 is mainly present in the proximal convoluted tubule, facilitating 90% of glucose reabsorption from the glomerular filtrate. SGLT2i, such as empagliflozin, dapagliflozin, canagliflozin, and ertugliflozin, block glucose reabsorption, thereby reducing blood glucose levels independently of insulin sensitivity and secretion [11]. GLP-1RA and DPP-4i control blood glucose by targeting the incretin system. The incretin hormones GLP-1 and gastric inhibitory peptide (GIP) are produced in the gastrointestinal system in response to nutrient intake. They stimulate insulin synthesis, suppress islet α-cell function, and promote the proliferation and differentiation of pancreas β-cells. Human GLP-1 is degraded within ~2–3 min in circulation, by DPP-4. DPP-4 inhibition increases levels of GLP-1 and GIP, which in turn promotes insulin secretion, thereby reducing the blood glucose [12]. GLP-1RA can be categorized as short-acting compounds, which provide short-lived receptor activation (exenatide and lixisenatide), or as long-acting compounds (albiglutide, dulaglutide, exenatide long-acting release, and liraglutide), which activate the GLP-1 receptor continuously [13]. The DPP4i sitagliptin, saxagliptin, linagliptin, alogliptin, and trelagliptin are approved by the FDA as well, while vildagliptin has approval only from the EMA.

**Figure 1:**
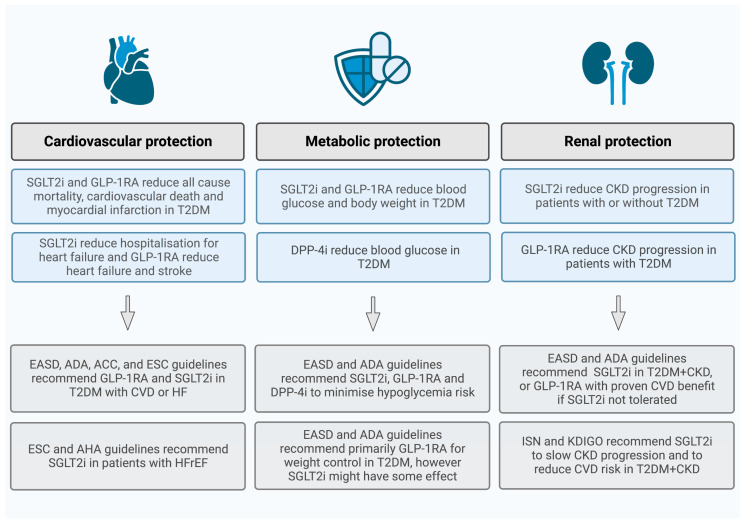
The protective effects of sodium-glucose cotransporter-2 inhibitors (SGLT2i), glucagon-like peptide-1 receptors agonists (GLP-1RA), and dipeptidyl-peptidase-4 inhibitors (DPP-4i) in patients with type 2 diabetes mellitus (T2DM). ACC: American College of Cardiology, ADA: American Diabetes Association, AHA: American Heart Association, CKD: chronic kidney disease, CVD: cardiovascular disease, EASD: European Association for the Study of Diabetes, ESC: European Society of Cardiology, ISN: International Society of Nephrology, KDIGO: Kidney Disease: Improving Global Outcomes.

Numerous cardiovascular outcomes trials (CVOTs) have shown the cardiovascular safety and efficacy of these novel compounds. They reduce major adverse cardiovascular events (MACE) independently of their anti-hyperglycemic properties [14,15,16,17,18,19,20,21]. These positive results represent a paradigm shift in DM management, from glycemic control alone to the simultaneous improvement of cardiovascular outcomes. Scientific guidelines now recommend the addition of an SGLT2i or a GLP-1RA, or even their combination, to metformin for patients in whom CVD, DKD, or heart failure (HF) predominates [22,23,24]. Moreover, SGLT2i have been implemented in the heart failure 2021 guidelines of the European Society of Cardiology in patients with heart failure with reduced ejection fraction (HFrEF), and also the 2022 guidelines of the American Heart Association for the treatment of heart failure made similar recommendations [25,26]. Concerning DPP-4i, their cardioprotective effect is still questionable. Despite the results of preclinical and small clinical studies suggesting that DPP-4i are promising candidates, CVOTs have reported no beneficial cardiovascular effects so far (Figure 1) [27,28].

The potential mechanisms of cardioprotection can be various. SGLT2i drugs might exert several molecular actions beyond lowering blood glucose levels: they inhibit inflammation and oxidative stress, improve cardiac metabolism and bioenergetics, alter adipokine and cytokine production, and reduce cardiac fibrosis and necrosis. SGLT2i also have hemodynamic effects: they improve ventricular loading conditions through a reduction in preload (secondary to natriuresis, osmotic diuresis) and afterload (reduction in blood pressure and improvement in vascular function), can exert anti-atherosclerotic properties, and reduce cardiac arrhythmias. GLP-1RA also improve blood pressure, body weight, dyslipidemia, oxidative stress, and endothelial dysfunction. DPP4i have the potential to be indirectly involved in reducing myocardial ischemia by protecting GLP-1 from degradation, thus GLP-1 receptors, abundantly expressed in renal and cardiovascular tissue, can be further activated [29,30,31,32,33].

This article summarizes the current literature on the molecular cardioprotective effects of SGLT2i, GLP-1RA, and DPP-4i. It reviews the relevant mechanisms of action, focusing on inflammation, oxidative stress, and fibrosis.

## 2. Inflammation

Hyperglycemia stimulates the secretion of pro-inflammatory cytokines in visceral adipocytes and cardiac cells, contributing to the development of a chronic low-grade inflammatory state and leading to insulin resistance. High glucose levels and dyslipidemia directly induce the upregulation and secretion of cytokines such as tumor necrosis factor-alpha (TNF-α), interleukins (IL-6, IL-1β), and C-reactive protein (CRP). These cytokines play a key role in the activation of the nuclear factor-kappa-B (NF-κB), resulting in prominent recruitment of leukocytes and monocytes, leading to myocardial inflammation [34,35]. Activation of NF-κB via TLR-4 leads to further downstream release of pro-inflammatory cytokines and monocyte chemoattractant protein-1 (MCP-1). Persistent chronic inflammation partially mediates structural and metabolic changes in the diabetic heart, including left ventricular (LV) hypertrophy, myocardial fibrosis, and abnormalities in calcium handling.

### 2.1. SGLT2i

In vitro studies have demonstrated the anti-inflammatory effects of SGLT2i through the direct reduction in inflammatory molecules of both rodent and human endothelial cells and macrophages. Dapagliflozin inhibited TLR-4 overexpression and NF-κB activation in lipopolysaccharide (LPS)-stimulated endothelial cells and modulated macrophage polarization to the anti-inflammatory M2 phenotype [36]. Similarly, canagliflozin prevented the elevation of IL-6 and the activation of NF-κB pathways in LPS-stimulated human coronary artery endothelial cells [37]. Empagliflozin reduced the expression of pro-inflammatory cytokine and chemokine release through the IKK/NF-κB/JAK2-STAT1/3, and MKK4/7-JNK pathways [38]. Activation of AMP-activated protein kinase (AMPK) reduces oxidative stress, mitochondrial dysfunction, inflammation, and preserves ventricular function during cardiac ischemia, diabetes, or pressure overload. Empagliflozin restored AMPK phosphorylation in primary cardiomyocytes of obese mice, while dapagliflozin prevented high glucose-induced endothelial cell dysfunction by increasing AMPK phosphorylation [39,40]. Similarly, canagliflozin inhibited IL-6 and MCP-1 via an AMPK-dependent mechanism [41]. Hyperglycemia triggers Nod-like receptor (NLR) family pyrin domain-containing 3 (NLRP3) overactivation, which promotes the maturation and release of IL-1β and IL-18. Canagliflozin inhibited TNF-α-induced IL-1β production and NLRP3 inflammasome activation in human coronary artery endothelial cells from donors with DM [42].

In vivo results also confirmed the above-mentioned mechanisms. Luseogliflozin reduced TNFα, IL-1β, IL-6, and intercellular adhesion molecule-1 (ICAM-1) expression in the aorta of diabetic ApoE^−/−^ mice [43]. In parallel, dapagliflozin treatment decreased circulating levels of NLRP3, IL-1β, and IL-18, as well as in the abdominal aorta of ApoE^−/−^ mice with DM and atherosclerosis [44]. Similar findings have been shown in BTBR ob/ob T2DM mice, where dapagliflozin reduced the activation of the NLRP3/ASC inflammasome and cardiac levels of IL-1β, IL-6, and TNF-α [45]. For T1DM there are much less data. Parallel to improved vascular function, empagliflozin diminished mRNA expression of pro-inflammatory genes in the aorta of streptozotocin (STZ)-induced T1DM rats. In the same model, our group showed that dapagliflozin reduced the left ventricular levels of IL-1β, IL-6, and TNF-α in diabetic rats, suggesting its anti-inflammatory potential [46].

The impact of hyperglycemia on susceptibility to myocardial ischemia/reperfusion injury (IRI) is multifaceted, including cardioprotection, neutral effects, or increased susceptibility [47]. Recent studies showed that dapagliflozin was protective in other CVD models, such as against myocardial IRI and dilated cardiomyopathy, by limiting the NLRP3 inflammasome activation [48,49]. Furthermore, empagliflozin reduced infarct size in an Slc5a2 deficient mouse model on a C57Bl/6N background, suggesting that it may have a cardioprotective effect independently of its glucose-lowering effect via SGLT2 [50]. These effects appear to be separate from the anti-hyperglycemic properties of SGLT2i.

Findings from clinical studies are conflicting. A recent report suggested that administration of empagliflozin for 12 months to diabetic patients reduced the blood hs-CRP levels compared to baseline or placebo [51]. Similarly, the CANOSSA trial demonstrated that the administration of canagliflozin for 12 months decreased the levels of hs-CRP after 3, 6, and 12 months in patients with chronic heart failure and DM [52]. In the DEFENCE study, 16-week-long dapagliflozin add-on therapy to metformin improved endothelial function, measured by flow-mediated dilation, in patients with inadequately controlled early-stage T2DM [53]. By contrast, in the EMBLEM trial in T2DM patients with established CVD, a 24-week treatment with empagliflozin did not improve endothelial dysfunction [54]. It is also well known from clinical studies that SGLT2i increase hematocrit and hemoglobin levels in T2DM, an effect that has been linked with cardiorenal protection, possibly by improving tissue oxygenization. Another plausible mechanism of SGLT2i’s anti-inflammatory effect has been suggested to be the suppression of hepcidin levels and an increase in erythropoiesis [55,56].

### 2.2. GLP-1RA

Several in vitro studies have demonstrated that liraglutide and exenatide can attenuate high glucose-induced cell death, TNF-α and IL-6 expression, and the activation of the NF-κB signaling pathway in cardiomyocytes [57,58]. In TNF-α-stimulated HUVECs, liraglutide has been shown to suppress NF-κB activation and the expression of various inflammatory factors, including vascular cell adhesion molecule-1 (VCAM-1), ICAM-1, E-selectin, and MCP-1 [59]. Furthermore, liraglutide has been found to inhibit the phosphorylation of NF-κB in a concentration-dependent manner, leading to the suppression of endothelin-1 expression in HUVECs [60]. It also induced AMPK activation, via increasing calmodulin-dependent protein kinase-β activation, in human aortic endothelial cells [61]. Dulaglutide has been found to inhibit NLRP3 inflammasome activation, which reduces the maturation and release of IL-1β, IL-8, and SIRT1 [62].

The cardioprotective effect of GLP-1RA has been investigated in a wide range of animal models. Liraglutide has been studied the most extensively. In STZ-induced diabetic rats, liraglutide decreased the levels of IL-1β, TNF-α, ICAM-1, and VCAM-1 in the ventricle [63,64]. In parallel, serum levels of IL-6 and IL-1β were decreased in liraglutide-treated diabetic non-human primates [65]. In a high-fat diet T2DM mice model, exendin-4 protected against heart remodeling and attenuated the levels of IL-1β, IL-6, TNF-α, ICAM-1, and VCAM-1 by inhibiting NLRP3 inflammasome-dependent inflammatory pathways [66,67]. In another model, using STZ-induced diabetic ApoE^−/−^ mice, GLP-1RA treatment reduced lipid deposition and plaque volume on the aortic surface [68]. Similar effects were observed in LDLr^−/−^ mice, where both liraglutide and semaglutide attenuated systemic inflammation and plaque lesion development [69]. GLP-1RA were also found to be protective in different non-diabetic murine models of cardiovascular diseases. NF-κB and MCP-1 expression was attenuated with liraglutide treatment in hypertensive mice [70]. Moreover, GLP-1RA reduced cardiac hypertrophy and protected against IRI via AMPK-dependent downstream pathways in different non-diabetic murine models [71,72,73].

Numerous CVOTs have shown the safety of GLP-1RA and the reduction in adverse cardiorenal outcomes in patients with T2DM [17,19,74]. The LIRAFLAME trial revealed that liraglutide reduced hs-CRP levels in patients with T2DM after 26 weeks [75]. Additional studies have shown that liraglutide and exenatide decrease the levels of IL-1β, IL-6, TNF-α, and CRP, and the risk of atherosclerosis formation [76,77,78].

### 2.3. DPP-4i

The effect of DPP-4i on diabetic cardiomyopathy remains a matter of debate. In vitro results have shown that trelagliptin treatment inhibited the expression of MCP-1, CXCL-1, IL-6, VCAM-1, and ICAM-1 in human aortic endothelial cells exposed to IL-1β, mimicking the microenvironment of atherosclerosis. Mechanistically, trelagliptin suppressed activation of the NF-κB pathway, which modulates the inflammatory signaling and monocyte adhesion [79]. Vildagliptin has also been shown to inhibit high-glucose-induced activation of NF-κB signaling and diminish TNF-α, IL-8, and MCP-1 in HAECs [80]. Similarly, vildagliptin reduced IL-1β, IL-6, TNF-α, TLR2, and TLR4 levels and suppressed the activation of the NF-κB pathway in LPS-stimulated murine macrophages [81]. Moreover, vildagliptin halted NLRP3 inflammasome activation and decreased IL-1β and IL-18 levels in free fatty acid-induced HUVECs [82].The anti-inflammatory properties of DPP-4i have been shown in different T1DM and T2DM animal models as well. Sitagliptin decreased serum levels of hs-CRP, IL-1β, MCP-1, and TNF-α in ZDF rats, while vildagliptin and linagliptin had a similar effect in high-fat diet-induced diabetic Wistar rats and western diet-induced diabetic C56Bl/6J mice [83,84,85]. In an STZ-induced T1DM diabetic Wistar rat model, sitagliptin also attenuated diabetic cardiomyopathy via the modulation of IL-6 levels and the JAK/STAT signaling pathway [86]. Linagliptin improved ejection fraction and decreased the levels of IL-1β and IL-6 by targeting the NLRP3/ASC inflammasome in db/db mice with myocardial infarction [87]. Interestingly, the addition of saxagliptin to dapagliflozin further reduced the activation of the NLRP3/ASC inflammasome in BTBR ob/ob T2DM mice [45]. In addition, alogliptin reduced atherosclerosis and inflammation, via inhibition of the monocyte activation/chemotaxis, in ApoE^−/−^ mice [88]. Recent data demonstrated that saxagliptin attenuated angiotensin II (AngII)-induced cardiac upregulation of NF-κB pathway activation, indicating that DPP-4i might have a cardioprotective effect independently of their glucose-lowering properties [89].

In a clinical trial, linagliptin reduced aortic pulse wave velocity, a surrogate marker for arterial stiffness and early atherosclerosis [27]. In another study, the addition of vildagliptin to metformin decreased the levels of IL-1β and hs-CRP in patients with T2DM and coronary artery disease [90]. Sitagliptin also decreased the levels of CRP in insulin-resistant newly diagnosed patients with T2DM [91]. In contrast, the sub-analysis from the REASON trial did not show significant changes either in hs-CRP or IL-6 in sitagliptin- or anagliptin-treated diabetic patients [92]. Collectively, it should be noted that the encouraging results from basic research and small clinical trials have not yet been fully translated into clinical evidence.

## 3. Oxidative Stress

Oxidative stress and chronic inflammation induce a switch of metabolic homeostasis, leading to decreased peripheral insulin sensitivity, β-cell dysfunction, and the development of diabetic cardiomyopathy. Hyperglycemia and lipotoxicity are associated with the increased production of reactive oxygen species (ROS) or reactive nitrogen species (RNS) in the mitochondria of cardiac myocytes, endothelial cells, and neutrophils. At the molecular level, excessive ROS production can worsen the formation of advanced glycation end-products (AGE), activation of the receptor for AGE (RAGE), and protein kinase C isoforms (PKC), increases the hexosamine pathway flux, and activates the NF-κB pathway [93]. These pathways not only directly contribute to cardiomyopathy but are themselves sources of additional ROS production [94].

### 3.1. SGLT2i

In vitro experiments mimicking diabetes by high glucose induction showed that empagliflozin alone, or combined with liraglutide, decreased cell death and oxidative stress via improved NOS activity and increased NO production in cardiac muscle cells [95]. In cardiomyoblasts, empagliflozin mitigated lipid-overload-induced ROS levels and improved mitochondrial membrane potential, partly via the activation of the nuclear factor erythroid 2-related factor 2 (Nrf2) signaling pathway [96]. Nrf2 is a key transcription factor for antioxidant and cytoprotective gene expression. Empagliflozin not only increased Nrf2 expression but also promoted its nuclear translocation [97]. SGLT2i are cytoprotective also in endothelial cells, both empagliflozin and dapagliflozin restore NO bioavailability by inhibiting ROS generation in TNFα-stimulated HUVECs and HCAECs [98,99].

A large body of animal experiments has confirmed the in vitro findings. A study has shown that empagliflozin can prevent cardiac dysfunction in diabetic db/db mice by improving oxidative stress and mitochondrial dysfunction [97]. Further studies have revealed the detailed mechanisms by which SGLT2i improve oxidative stress. In genetically modified T2DM KK-Ay mice, empagliflozin attenuated myocardial oxidative stress via the stimulation of Nrf2/ARE signaling and suppression of the TGF-β/Smad pathway [100]. Similar findings were observed in STZ-induced T1DM Sprague Dawley rats, where dapagliflozin reversed the upregulation of the TGF-β/Smad signaling [101]. Ertugliflozin has been found to modulate mitochondrial dysfunction and myocardial oxidative stress in high-fat, high-sucrose-fed C57BL/6J mice by preventing hydrogen peroxide release and preserving ATP production [102]. Canagliflozin has also been shown to moderate oxidative stress and reduced the expression of NADPH oxidase subunits such as NOX2, p22phox, p47phox, and the urinary excretion of 8-hydroxy-2′-deoxyguanosine (8-OHdG) [103].

There is only limited clinical data available, mainly due to the lack of specific biomarkers of oxidative stress. However, a recent clinical study has shown that plasma 8-iso-PGF_2*α*_ levels decreased after 24 weeks of dapagliflozin treatment in patients with newly diagnosed T2DM, referring to reduced oxidative stress [104]. In a pilot study, oxidative stress was evaluated by measuring the blood levels of soluble NADPH oxidase 2 (NOX2)-derivative peptide, which is a marker of NOX2 activation and hydrogen peroxide production in patients with T2DM. The study found that gliflozins significantly decreased the level of both markers [105].

### 3.2. GLP-1RA

In vitro experiments have shown that exendin-4 moderated high glucose-induced apoptosis and ROS production via the activation of the Nrf2 signaling pathway, increase the antioxidant capacity in rat cardiomyocytes [106]. As was mentioned above, liraglutide combined with empagliflozin has a synergistic effect in decreasing high glucose-induced cell death and oxidative stress in cardiomyocytes [95]. A novel oral GLP-1RA hypoglycemic peptide 2 (OHP2) and exendin-4 inhibited neutral lipid accumulation and intracellular and mitochondrial ROS generation in palmitate- or methylglyoxal-induced rat cardiomyocytes [107,108,109]. These effects were also confirmed in another model, where liraglutide ameliorated IL-1β-induced ROS production and NOX-4 expression in cardiomyocytes [110].

Evidence from in vivo diabetic models has shown the antioxidant effects of GLP-1RA. Exendin-4 ameliorated myocardial oxidative stress via the suppression of NOX-4, with concomitant elevation of superoxide dismutase 1 (SOD-1) and glutathione peroxidase, in genetic T2DM KK-Ay mice and high-fat diet-induced diabetic mice models [111]. Exenatide and OHP2 simultaneously attenuated cardiac ROS production by increasing the antioxidant enzymes manganese-dependent SOD and catalase in both STZ-induced T1DM and high-fat diet-induced T2DM rat models [109,112]. Additionally, liraglutide also decreased myocardial triglyceride and diacylglycerol levels, NOX activity, and oxidative stress, via the activation of the AMPK-Sirt1 pathway in STZ-induced T1DM rats [113].

In addition to basic research, a small clinical trial investigated the antioxidant effect of GLP-1RA. It was shown that plasma 8-iso-PGF_2*α*_ levels are reduced after 26 weeks of once-weekly dulaglutide injection in patients with T2DM, indicating decreased oxidative stress [114]. These data suggest that GLP-1RA exert antioxidant effects, by reducing ROS production and increasing the antioxidant capacity, partly independent of their glucose-lowering effect.

### 3.3. DPP-4i

There is limited research on the effect of DPP-4i in in vitro experiments. However, some studies have shown promising results. For instance, saxagliptin has been found to moderate hypoxia-induced cell death, ROS production, and NOX-4 expressions, while also rescuing mitochondrial membrane potential in rat cardiomyocytes [115]. In addition, sitagliptin reduced TNF-α and S100A12-induced cellular oxidative stress, NADPH oxidase, and NF-κB activation in human aortic smooth muscle cells. These findings suggest that sitagliptin may suppress the initiation and progression of arterial calcification by inhibiting the activation of these pathways [116].

Several in vivo studies have shown that DPP-4i attenuate cardiac and aortic oxidative stress in different diabetic animal models. Sitagliptin reduced cardiac oxidative stress, nitric oxide (NO), and malondialdehyde (MDA), a marker of antioxidant status, in diabetic ZDF (*fa*/*fa*) and STZ-induced Wistar rats [83,86]. Both sitagliptin and vildagliptin prevented cardiac mitochondrial ROS production, membrane depolarization, and swelling in high-fat diet-induced obese insulin-resistant Wistar rats [117]. These results were also confirmed in another model, where alogliptin alleviated mitochondrial ROS production and prevented mitochondrial membrane depolarization and swelling in alloxan-induced diabetic rabbits [118,119]. Administration of vildagliptin restored antioxidant activity and moderated the aortic levels of lipid peroxidation, catalase, and NADPH oxidase in high-fat diet and STZ-induced diabetic Swiss albino mice and diabetic OLETF rats [120,121]. Saxagliptin reversed increased myocardial lipid accumulation, oxidative stress, and apoptosis in high-fat diet-fed C57BL/6 diabetic mice [107]. Linagliptin normalized mitochondrial ultrastructure, although myocardial ROS and RNS production were not affected in Zucker obese rats [122].

## 4. Fibrosis

Both the aforementioned mechanisms, inflammation and oxidative stress, lead to cardiac fibrosis in the long run, by enhancing TGF-β signaling pathways, stimulating the transformation of fibroblasts to myofibroblasts, and promoting extracellular matrix (ECM) remodeling [123]. Myocardial fibrosis is characterized by elevated fibrillar collagen deposition, changes in the matrix metallopeptidase/tissue inhibitors of metallopeptidase ratio, and enhanced differentiation of cardiac fibroblast to myofibroblast [124]. Hyperglycemia contributes to fibrosis through the formation of advanced glycation end products and ROS, activating the cardiac immune response, and elevating the cardiac lipotoxicity [125,126,127]. Myocardial fibrosis is typically accompanied by cardiomyocyte hypertrophy and microvascular alterations, characterized by the thickening of the media of small intracardiac vessels [128]. Independent of other factors, fibrosis is associated with higher hospitalization rates for HF and with increased mortality in DM patients [129]. Treatments that directly target cardiac fibrosis are lacking; therefore, therapies that reverse fibrosis may provide a novel approach to halt the progression of HF in diabetic patients.

### 4.1. SGLT2i

Several lines of evidence suggest that SGLT2i may have favorable effect processes that underlie the structural left ventricular remodeling in DM. A recent in vitro study demonstrated that empagliflozin attenuated TGF-β1-induced fibroblast activation and ECM remodeling, and it suppressed profibrotic marker levels such as type-1 collagen, ACTA2, CTGF, fibronectin, and MMP-2 [130]. Another study revealed that luseogliflozin inhibited high glucose-induced TGF-β2 expression in mouse cardiomyocytes by suppressing NHE-1 activity [131]. Dapagliflozin also seems to be beneficial, as it inhibited high glucose-induced EMT via inhibition of TGF-β/Smad signaling in HUVECs, and directly inhibited proliferation, activation, and collagen production in rat primary cardiac fibroblasts [101].

The cardiac and peri-coronary antifibrotic effects of SGLT2i have been widely investigated in rodent models of DM. These beneficial effects appear to be independent of changes in blood pressure or glycemic control. Empagliflozin has been shown to ameliorate myocardial fibrosis by inhibiting the TGF-β/Smad pathway and activating Nrf2/ARE signaling in diabetic KK-Ay mice [100]. In more detail, empagliflozin reduced the levels of TGF-β1, Smad1, Smad2, and Smad3, as well as type I and III collagen, in the myocardium of diabetic mice. Dapagliflozin attenuated the activation of Nlrp3/ASC inflammasome, fibrosis, and deterioration of left ventricular ejection fraction in diabetic BTBR ob/ob mice [45]. Furthermore, according to our study, dapagliflozin is also cardioprotective via minimizing profibrotic growth factor elevation (TGF-β1, CTGF, PDGF) and left ventricular ECM remodeling in STZ-induced T1DM rats [46].

Recent reports suggest that SGLT2i may be involved in LV remodeling in diabetic patients. The placebo-controlled, randomized EMPA-HEART trial reported a reduction in LV mass indexed to the body surface, obtained with cardiac MRI after 6 months of treatment in patients with type 2 diabetes mellitus (T2DM), coronary artery disease, and preserved ejection fraction [132]. Another randomized trial showed that dapagliflozin treatment for 12 months significantly reduced LV mass, as measured by MRI in the ITT analysis in patients with T2DM [133]. Accordingly, one can postulate that SGLT2 inhibition, independent of hyperglycemia, has direct and favorable effects on cardiac fibroblast phenotype and function, which is one of the most important factors of heart failure.

### 4.2. GLP-1RA

In vitro experiments have investigated the antifibrotic effect of GLP-1RA treatment on cardiac fibroblasts exposed to high glucose. A recent study indicated that liraglutide inhibited high glucose-induced collagen formation via activation of the ERK/NF-κB pathway and F-actin degradation in primary cardiac fibroblasts [134]. Furthermore, liraglutide also attenuated high glucose-stimulated fibroblast activation, partly through the CD36-JNK-AP1 pathway [135]. Yu et al. revealed that liraglutide and exendin-4 attenuated glucose toxicity-induced cardiac injury through mTOR/ULK1-dependent autophagy [136].

The antifibrotic effect of GLP-1RA has also been shown in a wide range of rodent models. One study showed that liraglutide prevented age-induced interstitial cardiac fibrosis in C57BL/6J mice on a high-fat diet or a normal chow diet treated with AngII [70]. Other experimental data has also revealed that ECM accumulation and myocardial fibrosis were ameliorated in liraglutide-treated diabetic Wistar rats [63,137]. Exendin-4 reversed cardiac remodeling by normalizing cardiac steatosis and oxidative stress in diabetic KK-Ay mice [111]. Furthermore, exendin-4 protected against ECM remodeling and diastolic dysfunction in diabetic C57BL/6J mice via glucose-dependent modulation of paracrine communication between infiltrating macrophages and resident fibroblasts [138]. Exendin-4 also improved cardiac function and reduced monocyte infiltration, fibrosis, and apoptosis in mice with cardiac-specific MCP-1 overexpression [139]. Additionally, liraglutide attenuated cardiometabolic dysregulation and reduced cardiac hypertrophy, myocardial fibrosis, and natriuretic peptide levels in a novel C57BL/6J mice model of heart failure with preserved ejection fraction (HFpEF) [140]. These results indicate that targeting GLP-1 signaling can be a novel therapeutic strategy in diabetic cardiomyopathy.

### 4.3. DPP-4i

In vitro data on the antifibrotic effects of DPP-4i are very limited. One study showed that linagliptin markedly inhibited high glucose- and Ang II-induced collagen formation and F-actin degradation in cardiac fibroblasts via activation of the ERK/NF-κB pathway [134].

**Figure 2:**
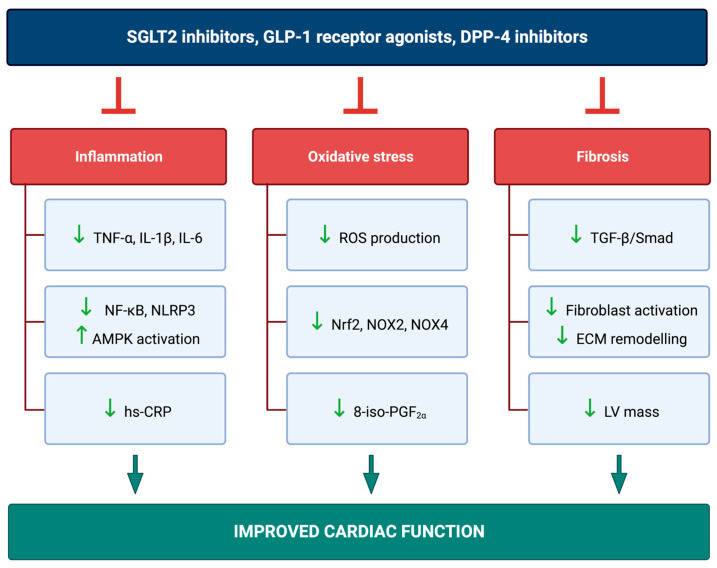
The effects of sodium-glucose cotransporter-2 inhibitors (SGLT2i), glucagon-like peptide-1 receptors agonists (GLP-1RA), and dipeptidyl-peptidase-4 inhibitors (DPP-4i) on inflammation, oxidative stress, and fibrosis. AMPK: adenosine monophosphate-activated protein kinase, ECM: extracellular matrix, hs-CRP: high-sensitivity C-reactive protein, IL-1β: interleukin-1 beta, IL-6: interleukin-6, LV: left ventricle, NF-κB: nuclear factor-κB, NLRP3: Nod-like receptor (NLR) family pyrin domain-containing 3, NOX2: NADPH oxidase 2, NOX4: NADPH oxidase 4, Nrf2: nuclear factor erythroid 2-related factor 2, ROS: reactive oxygen species, TGF-β: transforming growth factor β, TNF-α: tumor necrosis factor-alpha.

On the other hand, various in vivo studies investigated the effect of DPP-4i in different diabetic and obesity animal models. Sitagliptin reduced myocardial fibrosis, interstitial and perivascular collagen accumulation, and mRNA expression of TGF-β1 and CTGF in diabetic Goto–Kakizaki rats and Wistar rats [141,142]. Sitagliptin also prevented myocardial remodeling and decreased TGF-β1 protein levels in diabetic mice [143,144]. Alogliptin alleviated interstitial fibrosis and decreased atrial cardiomyocyte cross-sectional areas in diabetic rabbits [119]. Another study showed that gemigliptin decreased interstitial and perivascular fibrosis and cardiomyocyte cross-sectional areas in db/db mice [145]. The potent DPP-4i MK-0626 and linagliptin prevented diastolic dysfunction and reduced myocardial fibrosis in a mouse model of western diet-induced obesity [85,146]. Similarly, MK-0626 alleviated myocardial fibrosis by inhibiting TGF-β1 and Smad2/3 pathways in high-fat diet-induced obese rats [147]. Sitagliptin attenuated cardiac dysfunction and adverse remodeling following myocardial infarction in Fischer F344 diabetic rats [148]. Gu et al. also described similar findings, demonstrating that sitagliptin improved cardiac function and alleviated myocardial fibrosis after myocardial infarction, through activation of autophagy in diabetic C57BL/6 mice [149]. Vildagliptin decreased the LV expression of collagen genes and interstitial fibrosis in Dahl salt-sensitive rats [150]. Collectively, these experimental data suggest that DPP-4i may have antifibrotic properties (Figure 2).

## 5. Conclusions

The groundbreaking results of the clinical trials with SGLT2i and with GLP1-RA, and even with their combined use, have ensured cardiovascular safety and are likely to change the way that clinicians treat T2DM moving forward. Both SGLT2i and GLP1-RA have been found to reduce cardiovascular death and myocardial infarction. While SGLT2i prevent hospitalization for heart failure and show benefits across all left ventricular ejection fractions, GLP-1RA have the additional benefit of reducing stroke events compared to SGLT2i. In patients with established cardiovascular complications, SGLT2i can still be effective, particularly in those with heart failure, even without T2DM. However, it needs to be clarified whether these cardioprotective effects are specific to each drug or if they are universal class effects for the three different drug classes considered [151]. In addition to their preventive effect in T2DM, SGLT2i are also nephroprotective in non-diabetic patients, which further reduces cardiovascular complications, because CKD is a major risk factor for CVD. In conclusion, these new glucose-lowering drugs, especially SGLT2i and GLP1-RA, have opened a range of treatment options not only for T2DM but also for CKD and CVD, including their simultaneous presence in cardiorenal syndrome [29,152]. Elucidation of the pathomechanistic processes underlying the clinically seen beneficial effects of these drugs may improve understanding and influence further therapeutic applications.

## References

[1] Sun H., Saeedi P., Karuranga S., Pinkepank M., Ogurtsova K., Duncan B.B., Stein C., Basit A., Chan J.C.N., Mbanya J.C. (2022). IDF Diabetes Atlas: Global, regional and country-level diabetes prevalence estimates for 2021 and projections for 2045. Diabetes Res. Clin. Pract..

[2] Sarwar N., Gao P., Seshasai S.R., Gobin R., Kaptoge S., Di Angelantonio E., Ingelsson E., Lawlor D.A., Selvin E., Emerging Risk Factors Collaboration (2010). Diabetes mellitus, fasting blood glucose concentration, and risk of vascular disease: A collaborative meta-analysis of 102 prospective studies. Lancet.

[3] Sun D., Zhou T., Heianza Y., Li X., Fan M., Fonseca V.A., Qi L. (2019). Type 2 Diabetes and Hypertension. Circ. Res..

[4] Cherney D.Z.I., Repetto E., Wheeler D.C., Arnold S.V., MacLachlan S., Hunt P.R., Chen H., Vora J., Kosiborod M. (2020). Impact of Cardio-Renal-Metabolic Comorbidities on Cardiovascular Outcomes and Mortality in Type 2 Diabetes Mellitus. Am. J. Nephrol..

[5] Rao Kondapally Seshasai S., Kaptoge S., Thompson A., Di Angelantonio E., Gao P., Sarwar N., Whincup P.H., Mukamal K.J., Gillum R.F., Holme I. (2011). Diabetes mellitus, fasting glucose, and risk of cause-specific death. N. Engl. J. Med..

[6] Shah A.D., Langenberg C., Rapsomaniki E., Denaxas S., Pujades-Rodriguez M., Gale C.P., Deanfield J., Smeeth L., Timmis A., Hemingway H. (2015). Type 2 diabetes and incidence of cardiovascular diseases: A cohort study in 1.9 million people. Lancet Diabetes Endocrinol..

[7] Einarson T.R., Acs A., Ludwig C., Panton U.H. (2018). Prevalence of cardiovascular disease in type 2 diabetes: A systematic literature review of scientific evidence from across the world in 2007-2017. Cardiovasc. Diabetol..

[8] Ma C.X., Ma X.N., Guan C.H., Li Y.D., Mauricio D., Fu S.B. (2022). Cardiovascular disease in type 2 diabetes mellitus: Progress toward personalized management. Cardiovasc. Diabetol..

[9] Hiatt W.R., Kaul S., Smith R.J. (2013). The cardiovascular safety of diabetes drugs—Insights from the rosiglitazone experience. N. Engl. J. Med..

[10] US Food and Drug Administration (2020). Type 2 Diabetes Mellitus: Evaluating the Safety of New Drugs for Improving Glycemic Control Guidance for Industry.

[11] Wright E.M., Loo D.D., Hirayama B.A. (2011). Biology of human sodium glucose transporters. Physiol. Rev..

[12] Capuano A., Sportiello L., Maiorino M.I., Rossi F., Giugliano D., Esposito K. (2013). Dipeptidyl peptidase-4 inhibitors in type 2 diabetes therapy—Focus on alogliptin. Drug Des. Devel. Ther..

[13] Meier J.J. (2012). GLP-1 receptor agonists for individualized treatment of type 2 diabetes mellitus. Nat. Rev. Endocrinol..

[14] Zinman B., Wanner C., Lachin J.M., Fitchett D., Bluhmki E., Hantel S., Mattheus M., Devins T., Johansen O.E., Woerle H.J. (2015). Empagliflozin, Cardiovascular Outcomes, and Mortality in Type 2 Diabetes. N. Engl. J. Med..

[15] Neal B., Perkovic V., Mahaffey K.W., de Zeeuw D., Fulcher G., Erondu N., Shaw W., Law G., Desai M., Matthews D.R. (2017). Canagliflozin and Cardiovascular and Renal Events in Type 2 Diabetes. N. Engl. J. Med..

[16] Wiviott S.D., Raz I., Bonaca M.P., Mosenzon O., Kato E.T., Cahn A., Silverman M.G., Zelniker T.A., Kuder J.F., Murphy S.A. (2019). Dapagliflozin and Cardiovascular Outcomes in Type 2 Diabetes. N. Engl. J. Med..

[17] Marso S.P., Daniels G.H., Brown-Frandsen K., Kristensen P., Mann J.F., Nauck M.A., Nissen S.E., Pocock S., Poulter N.R., Ravn L.S. (2016). Liraglutide and Cardiovascular Outcomes in Type 2 Diabetes. N. Engl. J. Med..

[18] Marso S.P., Bain S.C., Consoli A., Eliaschewitz F.G., Jodar E., Leiter L.A., Lingvay I., Rosenstock J., Seufert J., Warren M.L. (2016). Semaglutide and Cardiovascular Outcomes in Patients with Type 2 Diabetes. N. Engl. J. Med..

[19] Gerstein H.C., Colhoun H.M., Dagenais G.R., Diaz R., Lakshmanan M., Pais P., Probstfield J., Riesmeyer J.S., Riddle M.C., Ryden L. (2019). Dulaglutide and cardiovascular outcomes in type 2 diabetes (REWIND): A double-blind, randomised placebo-controlled trial. Lancet.

[20] Hernandez A.F., Green J.B., Janmohamed S., D’Agostino R.B., Granger C.B., Jones N.P., Leiter L.A., Rosenberg A.E., Sigmon K.N., Somerville M.C. (2018). Albiglutide and cardiovascular outcomes in patients with type 2 diabetes and cardiovascular disease (Harmony Outcomes): A double-blind, randomised placebo-controlled trial. Lancet.

[21] Cannon C.P., Pratley R., Dagogo-Jack S., Mancuso J., Huyck S., Masiukiewicz U., Charbonnel B., Frederich R., Gallo S., Cosentino F. (2020). Cardiovascular Outcomes with Ertugliflozin in Type 2 Diabetes. N. Engl. J. Med..

[22] Cosentino F., Grant P.J., Aboyans V., Bailey C.J., Ceriello A., Delgado V., Federici M., Filippatos G., Grobbee D.E., Hansen T.B. (2020). 2019 ESC Guidelines on diabetes, pre-diabetes, and cardiovascular diseases developed in collaboration with the EASD. Eur. Heart J..

[23] Kidney Disease: Improving Global Outcomes (KDIGO) Diabetes Work Group (2020). KDIGO 2020 Clinical Practice Guideline for Diabetes Management in Chronic Kidney Disease. Kidney Int..

[24] Draznin B., Aroda V.R., Bakris G., Benson G., Brown F.M., Freeman R., Green J., Huang E., Isaacs D., American Diabetes Association Professional Practice Committee (2022). 9. Pharmacologic Approaches to Glycemic Treatment: Standards of Medical Care in Diabetes-2022. Diabetes Care.

[25] McDonagh T.A., Metra M., Adamo M., Gardner R.S., Baumbach A., Bohm M., Burri H., Butler J., Celutkiene J., Chioncel O. (2021). 2021 ESC Guidelines for the diagnosis and treatment of acute and chronic heart failure. Eur. Heart J..

[26] Heidenreich P.A., Bozkurt B., Aguilar D., Allen L.A., Byun J.J., Colvin M.M., Deswal A., Drazner M.H., Dunlay S.M., Evers L.R. (2022). 2022 AHA/ACC/HFSA Guideline for the Management of Heart Failure: A Report of the American College of Cardiology/American Heart Association Joint Committee on Clinical Practice Guidelines. Circulation.

[27] De Boer S.A., Heerspink H.J.L., Juarez Orozco L.E., van Roon A.M., Kamphuisen P.W., Smit A.J., Slart R., Lefrandt J.D., Mulder D.J. (2017). Effect of linagliptin on pulse wave velocity in early type 2 diabetes: A randomized, double-blind, controlled 26-week trial (RELEASE). Diabetes Obes. Metab..

[28] Jax T., Stirban A., Terjung A., Esmaeili H., Berk A., Thiemann S., Chilton R., von Eynatten M., Marx N. (2017). A randomised, active- and placebo-controlled, three-period crossover trial to investigate short-term effects of the dipeptidyl peptidase-4 inhibitor linagliptin on macro- and microvascular endothelial function in type 2 diabetes. Cardiovasc. Diabetol..

[29] Braunwald E. (2022). Gliflozins in the Management of Cardiovascular Disease. N. Engl. J. Med..

[30] Verma S., McMurray J.J.V. (2018). SGLT2 inhibitors and mechanisms of cardiovascular benefit: A state-of-the-art review. Diabetologia.

[31] Pahud de Mortanges A., Salvador D., Laimer M., Muka T., Wilhelm M., Bano A. (2021). The Role of SGLT2 Inhibitors in Atherosclerosis: A Narrative Mini-Review. Front. Pharmacol..

[32] Kolesnik E., Scherr D., Rohrer U., Benedikt M., Manninger M., Sourij H., von Lewinski D. (2022). SGLT2 Inhibitors and Their Antiarrhythmic Properties. Int. J. Mol. Sci..

[33] Panda S.P. (2023). Role of DPP4 and DPP4i in Glucose Homeostasis and Cardiorenal Syndrome. Endocr. Metab. Immune. Disord. Drug Targets.

[34] Maier H.J., Schips T.G., Wietelmann A., Kruger M., Brunner C., Sauter M., Klingel K., Bottger T., Braun T., Wirth T. (2012). Cardiomyocyte-specific IkappaB kinase (IKK)/NF-kappaB activation induces reversible inflammatory cardiomyopathy and heart failure. Proc. Natl. Acad. Sci. USA.

[35] Frieler R.A., Mortensen R.M. (2015). Immune cell and other noncardiomyocyte regulation of cardiac hypertrophy and remodeling. Circulation.

[36] Abdollahi E., Keyhanfar F., Delbandi A.A., Falak R., Hajimiresmaiel S.J., Shafiei M. (2022). Dapagliflozin exerts anti-inflammatory effects via inhibition of LPS-induced TLR-4 overexpression and NF-kappaB activation in human endothelial cells and differentiated macrophages. Eur. J. Pharmacol..

[37] Uthman L., Kuschma M., Romer G., Boomsma M., Kessler J., Hermanides J., Hollmann M.W., Preckel B., Zuurbier C.J., Weber N.C. (2021). Novel Anti-inflammatory Effects of Canagliflozin Involving Hexokinase II in Lipopolysaccharide-Stimulated Human Coronary Artery Endothelial Cells. Cardiovasc. Drugs Ther..

[38] Lee N., Heo Y.J., Choi S.E., Jeon J.Y., Han S.J., Kim D.J., Kang Y., Lee K.W., Kim H.J. (2021). Anti-inflammatory Effects of Empagliflozin and Gemigliptin on LPS-Stimulated Macrophage via the IKK/NF-kappaB, MKK7/JNK, and JAK2/STAT1 Signalling Pathways. J. Immunol. Res..

[39] Sun X., Han F., Lu Q., Li X., Ren D., Zhang J., Han Y., Xiang Y.K., Li J. (2020). Empagliflozin Ameliorates Obesity-Related Cardiac Dysfunction by Regulating Sestrin2-Mediated AMPK-mTOR Signaling and Redox Homeostasis in High-Fat Diet-Induced Obese Mice. Diabetes.

[40] Faridvand Y., Kazemzadeh H., Vahedian V., Mirzajanzadeh P., Nejabati H.R., Safaie N., Maroufi N.F., Pezeshkian M., Nouri M., Jodati A. (2022). Dapagliflozin attenuates high glucose-induced endothelial cell apoptosis and inflammation through AMPK/SIRT1 activation. Clin. Exp. Pharmacol. Physiol..

[41] Feijoo-Bandin S., Aragon-Herrera A., Otero-Santiago M., Anido-Varela L., Morana-Fernandez S., Tarazon E., Rosello-Lleti E., Portoles M., Gualillo O., Gonzalez-Juanatey J.R. (2022). Role of Sodium-Glucose Co-Transporter 2 Inhibitors in the Regulation of Inflammatory Processes in Animal Models. Int. J. Mol. Sci..

[42] Li X., Kerindongo R.P., Preckel B., Kalina J.O., Hollmann M.W., Zuurbier C.J., Weber N.C. (2023). Canagliflozin inhibits inflammasome activation in diabetic endothelial cells—Revealing a novel calcium-dependent anti-inflammatory effect of canagliflozin on human diabetic endothelial cells. Biomed. Pharmacother..

[43] Nakatsu Y., Kokubo H., Bumdelger B., Yoshizumi M., Yamamotoya T., Matsunaga Y., Ueda K., Inoue Y., Inoue M.K., Fujishiro M. (2017). The SGLT2 Inhibitor Luseogliflozin Rapidly Normalizes Aortic mRNA Levels of Inflammation-Related but Not Lipid-Metabolism-Related Genes and Suppresses Atherosclerosis in Diabetic ApoE KO Mice. Int. J. Mol. Sci..

[44] Leng W., Ouyang X., Lei X., Wu M., Chen L., Wu Q., Deng W., Liang Z. (2016). The SGLT-2 Inhibitor Dapagliflozin Has a Therapeutic Effect on Atherosclerosis in Diabetic ApoE(-/-) Mice. Mediat. Inflamm..

[45] Ye Y., Bajaj M., Yang H.C., Perez-Polo J.R., Birnbaum Y. (2017). SGLT-2 Inhibition with Dapagliflozin Reduces the Activation of the Nlrp3/ASC Inflammasome and Attenuates the Development of Diabetic Cardiomyopathy in Mice with Type 2 Diabetes. Further Augmentation of the Effects with Saxagliptin, a DPP4 Inhibitor. Cardiovasc. Drugs Ther..

[46] Hodrea J., Saeed A., Molnar A., Fintha A., Barczi A., Wagner L.J., Szabo A.J., Fekete A., Balogh D.B. (2022). SGLT2 inhibitor dapagliflozin prevents atherosclerotic and cardiac complications in experimental type 1 diabetes. PLoS ONE.

[47] Penna C., Andreadou I., Aragno M., Beauloye C., Bertrand L., Lazou A., Falcao-Pires I., Bell R., Zuurbier C.J., Pagliaro P. (2020). Effect of hyperglycaemia and diabetes on acute myocardial ischaemia-reperfusion injury and cardioprotection by ischaemic conditioning protocols. Br. J. Pharmacol..

[48] Yu Y.W., Que J.Q., Liu S., Huang K.Y., Qian L., Weng Y.B., Rong F.N., Wang L., Zhou Y.Y., Xue Y.J. (2021). Sodium-Glucose Co-transporter-2 Inhibitor of Dapagliflozin Attenuates Myocardial Ischemia/Reperfusion Injury by Limiting NLRP3 Inflammasome Activation and Modulating Autophagy. Front. Cardiovasc. Med..

[49] Hu J., Xu J., Tan X., Li D., Yao D., Xu B., Lei Y. (2023). Dapagliflozin protects against dilated cardiomyopathy progression by targeting NLRP3 inflammasome activation. Naunyn. Schmiedebergs Arch. Pharmacol..

[50] Chen S., Wang Q., Christodoulou A., Mylonas N., Bakker D., Nederlof R., Hollmann M.W., Weber N.C., Coronel R., Wakker V. (2023). Sodium Glucose Cotransporter-2 Inhibitor Empagliflozin Reduces Infarct Size Independently of Sodium Glucose Cotransporter-2. Circulation.

[51] Hattori S. (2018). Anti-inflammatory effects of empagliflozin in patients with type 2 diabetes and insulin resistance. Diabetol. Metab. Syndr..

[52] Sezai A., Sekino H., Unosawa S., Taoka M., Osaka S., Tanaka M. (2019). Canagliflozin for Japanese patients with chronic heart failure and type II diabetes. Cardiovasc. Diabetol..

[53] Shigiyama F., Kumashiro N., Miyagi M., Ikehara K., Kanda E., Uchino H., Hirose T. (2017). Effectiveness of dapagliflozin on vascular endothelial function and glycemic control in patients with early-stage type 2 diabetes mellitus: DEFENCE study. Cardiovasc. Diabetol..

[54] Tanaka A., Shimabukuro M., Machii N., Teragawa H., Okada Y., Shima K.R., Takamura T., Taguchi I., Hisauchi I., Toyoda S. (2019). Effect of Empagliflozin on Endothelial Function in Patients With Type 2 Diabetes and Cardiovascular Disease: Results from the Multicenter, Randomized, Placebo-Controlled, Double-Blind EMBLEM Trial. Diabetes Care.

[55] Kanbay M., Tapoi L., Ureche C., Tanriover C., Cevik E., Demiray A., Afsar B., Cherney D.Z.I., Covic A. (2022). Effect of sodium-glucose cotransporter 2 inhibitors on hemoglobin and hematocrit levels in type 2 diabetes: A systematic review and meta-analysis. Int. Urol. Nephrol..

[56] Ghanim H., Abuaysheh S., Hejna J., Green K., Batra M., Makdissi A., Chaudhuri A., Dandona P. (2020). Dapagliflozin Suppresses Hepcidin And Increases Erythropoiesis. J. Clin. Endocrinol. Metab..

[57] Ma G., Liu Y., Wang Y., Wen Z., Li X., Zhai H., Miao L., Luo J. (2020). Liraglutide reduces hyperglycemia-induced cardiomyocyte death through activating glucagon-like peptide 1 receptor and targeting AMPK pathway. J. Recept. Signal Transduct. Res..

[58] Fu Z., Mui D., Zhu H., Zhang Y. (2020). Exenatide inhibits NF-kappaB and attenuates ER stress in diabetic cardiomyocyte models. Aging.

[59] Hattori Y., Jojima T., Tomizawa A., Satoh H., Hattori S., Kasai K., Hayashi T. (2010). A glucagon-like peptide-1 (GLP-1) analogue, liraglutide, upregulates nitric oxide production and exerts anti-inflammatory action in endothelial cells. Diabetologia.

[60] Dai Y., Mehta J.L., Chen M. (2013). Glucagon-like peptide-1 receptor agonist liraglutide inhibits endothelin-1 in endothelial cell by repressing nuclear factor-kappa B activation. Cardiovasc. Drugs Ther..

[61] Krasner N.M., Ido Y., Ruderman N.B., Cacicedo J.M. (2014). Glucagon-like peptide-1 (GLP-1) analog liraglutide inhibits endothelial cell inflammation through a calcium and AMPK dependent mechanism. PLoS ONE.

[62] Luo X., Hu Y., He S., Ye Q., Lv Z., Liu J., Chen X. (2019). Dulaglutide inhibits high glucose- induced endothelial dysfunction and NLRP3 inflammasome activation. Arch. Biochem. Biophys..

[63] Trang N.N., Chung C.C., Lee T.W., Cheng W.L., Kao Y.H., Huang S.Y., Lee T.I., Chen Y.J. (2021). Empagliflozin and Liraglutide Differentially Modulate Cardiac Metabolism in Diabetic Cardiomyopathy in Rats. Int. J. Mol. Sci..

[64] Baylan U., Korn A., Emmens R.W., Schalkwijk C.G., Niessen H.W.M., Krijnen P.A.J., Simsek S. (2022). Liraglutide treatment attenuates inflammation markers in the cardiac, cerebral and renal microvasculature in streptozotocin-induced diabetic rats. Eur. J. Clin. Investig..

[65] Navabi R., Negahdari B., Hajizadeh-Saffar E., Hajinasrollah M., Jenab Y., Rabbani S., Pakzad M., Hassani S.N., Hezavehei M., Jafari-Atrabi M. (2021). Combined therapy of mesenchymal stem cells with a GLP-1 receptor agonist, liraglutide, on an inflammatory-mediated diabetic non-human primate model. Life Sci..

[66] Wei H., Bu R., Yang Q., Jia J., Li T., Wang Q., Chen Y. (2019). Exendin-4 Protects against Hyperglycemia-Induced Cardiomyocyte Pyroptosis via the AMPK-TXNIP Pathway. J. Diabetes Res..

[67] Zhang Z., Wang X., Yang L., Yang L., Ma H. (2021). Liraglutide ameliorates myocardial damage in experimental diabetic rats by inhibiting pyroptosis via Sirt1/AMPK signaling. Iran. J. Basic Med. Sci..

[68] Koshibu M., Mori Y., Saito T., Kushima H., Hiromura M., Terasaki M., Takada M., Fukui T., Hirano T. (2019). Antiatherogenic effects of liraglutide in hyperglycemic apolipoprotein E-null mice via AMP-activated protein kinase-independent mechanisms. Am. J. Physiol. Endocrinol. Metab..

[69] Rakipovski G., Rolin B., Nohr J., Klewe I., Frederiksen K.S., Augustin R., Hecksher-Sorensen J., Ingvorsen C., Polex-Wolf J., Knudsen L.B. (2018). The GLP-1 Analogs Liraglutide and Semaglutide Reduce Atherosclerosis in ApoE(-/-) and LDLr(-/-) Mice by a Mechanism That Includes Inflammatory Pathways. JACC Basic Transl. Sci..

[70] Gaspari T., Brdar M., Lee H.W., Spizzo I., Hu Y., Widdop R.E., Simpson R.W., Dear A.E. (2016). Molecular and cellular mechanisms of glucagon-like peptide-1 receptor agonist-mediated attenuation of cardiac fibrosis. Diab. Vasc. Dis. Res..

[71] Eid R.A., Bin-Meferij M.M., El-Kott A.F., Eleawa S.M., Zaki M.S.A., Al-Shraim M., El-Sayed F., Eldeen M.A., Alkhateeb M.A., Alharbi S.A. (2021). Exendin-4 Protects Against Myocardial Ischemia-Reperfusion Injury by Upregulation of SIRT1 and SIRT3 and Activation of AMPK. J. Cardiovasc. Transl. Res..

[72] Ma Z.G., Dai J., Zhang W.B., Yuan Y., Liao H.H., Zhang N., Bian Z.Y., Tang Q.Z. (2016). Protection against cardiac hypertrophy by geniposide involves the GLP-1 receptor/AMPKalpha signalling pathway. Br. J. Pharmacol..

[73] Zhou Y., He X., Chen Y., Huang Y., Wu L., He J. (2015). Exendin-4 attenuates cardiac hypertrophy via AMPK/mTOR signaling pathway activation. Biochem. Biophys. Res. Commun..

[74] Holman R.R., Bethel M.A., Mentz R.J., Thompson V.P., Lokhnygina Y., Buse J.B., Chan J.C., Choi J., Gustavson S.M., Iqbal N. (2017). Effects of Once-Weekly Exenatide on Cardiovascular Outcomes in Type 2 Diabetes. N. Engl. J. Med..

[75] Jensen J.K., Zobel E.H., von Scholten B.J., Rotbain Curovic V., Hansen T.W., Rossing P., Kjaer A., Ripa R.S. (2021). Effect of 26 Weeks of Liraglutide Treatment on Coronary Artery Inflammation in Type 2 Diabetes Quantified by [(64)Cu]Cu-DOTATATE PET/CT: Results from the LIRAFLAME Trial. Front. Endocrinol..

[76] Nauck M.A., Meier J.J., Cavender M.A., Abd El Aziz M., Drucker D.J. (2017). Cardiovascular Actions and Clinical Outcomes With Glucagon-Like Peptide-1 Receptor Agonists and Dipeptidyl Peptidase-4 Inhibitors. Circulation.

[77] Daousi C., Pinkney J.H., Cleator J., Wilding J.P., Ranganath L.R. (2013). Acute peripheral administration of synthetic human GLP-1 (7-36 amide) decreases circulating IL-6 in obese patients with type 2 diabetes mellitus: A potential role for GLP-1 in modulation of the diabetic pro-inflammatory state?. Regul. Pept..

[78] Lee Y.S., Jun H.S. (2016). Anti-Inflammatory Effects of GLP-1-Based Therapies beyond Glucose Control. Mediat. Inflamm..

[79] Meng J., Zhang W., Wang C., Xiong S., Wang Q., Li H., Liu G., Hao Z. (2020). The dipeptidyl peptidase (DPP)-4 inhibitor trelagliptin inhibits IL-1beta-induced endothelial inflammation and monocytes attachment. Int. Immunopharmacol..

[80] Wicinski M., Gorski K., Wodkiewicz E., Walczak M., Nowaczewska M., Malinowski B. (2020). Vasculoprotective Effects of Vildagliptin. Focus on Atherogenesis. Int. J. Mol. Sci..

[81] Lee D.S., Lee E.S., Alam M.M., Jang J.H., Lee H.S., Oh H., Kim Y.C., Manzoor Z., Koh Y.S., Kang D.G. (2016). Soluble DPP-4 up-regulates toll-like receptors and augments inflammatory reactions, which are ameliorated by vildagliptin or mannose-6-phosphate. Metabolism.

[82] Qi Y., Du X., Yao X., Zhao Y. (2019). Vildagliptin inhibits high free fatty acid (FFA)-induced NLRP3 inflammasome activation in endothelial cells. Artif. Cells Nanomed. Biotechnol..

[83] Ferreira L., Teixeira-de-Lemos E., Pinto F., Parada B., Mega C., Vala H., Pinto R., Garrido P., Sereno J., Fernandes R. (2010). Effects of sitagliptin treatment on dysmetabolism, inflammation, and oxidative stress in an animal model of type 2 diabetes (ZDF rat). Mediat. Inflamm..

[84] Tanajak P., Sa-Nguanmoo P., Apaijai N., Wang X., Liang G., Li X., Jiang C., Chattipakorn S.C., Chattipakorn N. (2017). Comparisons of cardioprotective efficacy between fibroblast growth factor 21 and dipeptidyl peptidase-4 inhibitor in prediabetic rats. Cardiovasc. Ther..

[85] Aroor A.R., Habibi J., Kandikattu H.K., Garro-Kacher M., Barron B., Chen D., Hayden M.R., Whaley-Connell A., Bender S.B., Klein T. (2017). Dipeptidyl peptidase-4 (DPP-4) inhibition with linagliptin reduces western diet-induced myocardial TRAF3IP2 expression, inflammation and fibrosis in female mice. Cardiovasc. Diabetol..

[86] Al-Rasheed N.M., Al-Rasheed N.M., Hasan I.H., Al-Amin M.A., Al-Ajmi H.N., Mahmoud A.M. (2016). Sitagliptin attenuates cardiomyopathy by modulating the JAK/STAT signaling pathway in experimental diabetic rats. Drug Des. Devel. Ther..

[87] Birnbaum Y., Tran D., Bajaj M., Ye Y. (2019). DPP-4 inhibition by linagliptin prevents cardiac dysfunction and inflammation by targeting the Nlrp3/ASC inflammasome. Basic Res. Cardiol..

[88] Shah Z., Kampfrath T., Deiuliis J.A., Zhong J., Pineda C., Ying Z., Xu X., Lu B., Moffatt-Bruce S., Durairaj R. (2011). Chronic DPP-4 Inhibition Reduces Atherosclerosis and Inflammation via Effects on Monocyte Recruitment and Chemotaxis. Circulation.

[89] Brown S.M., Smith C.E., Meuth A.I., Khan M., Aroor A.R., Cleeton H.M., Meininger G.A., Sowers J.R., DeMarco V.G., Chandrasekar B. (2017). Dipeptidyl Peptidase-4 Inhibition With Saxagliptin Ameliorates Angiotensin II-Induced Cardiac Diastolic Dysfunction in Male Mice. Endocrinology.

[90] Younis A., Eskenazi D., Goldkorn R., Leor J., Naftali-Shani N., Fisman E.Z., Tenenbaum A., Goldenberg I., Klempfner R. (2017). The addition of vildagliptin to metformin prevents the elevation of interleukin 1ss in patients with type 2 diabetes and coronary artery disease: A prospective, randomized, open-label study. Cardiovasc. Diabetol..

[91] Sun Y., Yan D., Hao Z., Cui L., Li G. (2020). Effects of Dapagliflozin and Sitagliptin on Insulin Resistant and Body Fat Distribution in Newly Diagnosed Type 2 Diabetic Patients. Med. Sci. Monit..

[92] Teragawa H., Morimoto T., Fujii Y., Ueda T., Sakuma M., Shimabukuro M., Arasaki O., Node K., Nomiyama T., Ueda S. (2020). Effect of Anagliptin versus Sitagliptin on Inflammatory Markers: Sub-Analysis from the REASON Trial. Diabetes Metab. Syndr. Obes..

[93] Evans J.L., Goldfine I.D., Maddux B.A., Grodsky G.M. (2002). Oxidative stress and stress-activated signaling pathways: A unifying hypothesis of type 2 diabetes. Endocr. Rev..

[94] Liu Q., Wang S., Cai L. (2014). Diabetic cardiomyopathy and its mechanisms: Role of oxidative stress and damage. J. Diabetes Investig..

[95] Cessario J., Pierre-Louis V., Wahl J., Li Z. (2021). Empagliflozin, alone or in combination with liraglutide, limits cell death in vitro: Role of oxidative stress and nitric oxide. Pharmacol. Rep..

[96] Bugga P., Mohammed S.A., Alam M.J., Katare P., Meghwani H., Maulik S.K., Arava S., Banerjee S.K. (2022). Empagliflozin prohibits high-fructose diet-induced cardiac dysfunction in rats via attenuation of mitochondria-driven oxidative stress. Life Sci..

[97] Wang J., Huang X., Liu H., Chen Y., Li P., Liu L., Li J., Ren Y., Huang J., Xiong E. (2022). Empagliflozin Ameliorates Diabetic Cardiomyopathy via Attenuating Oxidative Stress and Improving Mitochondrial Function. Oxid. Med. Cell. Longev..

[98] Uthman L., Homayr A., Juni R.P., Spin E.L., Kerindongo R., Boomsma M., Hollmann M.W., Preckel B., Koolwijk P., van Hinsbergh V.W.M. (2019). Empagliflozin and Dapagliflozin Reduce ROS Generation and Restore NO Bioavailability in Tumor Necrosis Factor alpha-Stimulated Human Coronary Arterial Endothelial Cells. Cell. Physiol. Biochem..

[99] Uthman L., Li X., Baartscheer A., Schumacher C.A., Baumgart P., Hermanides J., Preckel B., Hollmann M.W., Coronel R., Zuurbier C.J. (2022). Empagliflozin reduces oxidative stress through inhibition of the novel inflammation/NHE/[Na(+)](c)/ROS-pathway in human endothelial cells. Biomed. Pharmacother..

[100] Li C., Zhang J., Xue M., Li X., Han F., Liu X., Xu L., Lu Y., Cheng Y., Li T. (2019). SGLT2 inhibition with empagliflozin attenuates myocardial oxidative stress and fibrosis in diabetic mice heart. Cardiovasc. Diabetol..

[101] Tian J., Zhang M., Suo M., Liu D., Wang X., Liu M., Pan J., Jin T., An F. (2021). Dapagliflozin alleviates cardiac fibrosis through suppressing EndMT and fibroblast activation via AMPKalpha/TGF-beta/Smad signalling in type 2 diabetic rats. J. Cell. Mol. Med..

[102] Croteau D., Luptak I., Chambers J.M., Hobai I., Panagia M., Pimentel D.R., Siwik D.A., Qin F., Colucci W.S. (2021). Effects of Sodium-Glucose Linked Transporter 2 Inhibition With Ertugliflozin on Mitochondrial Function, Energetics, and Metabolic Gene Expression in the Presence and Absence of Diabetes Mellitus in Mice. J. Am. Heart Assoc..

[103] Rahadian A., Fukuda D., Salim H.M., Yagi S., Kusunose K., Yamada H., Soeki T., Sata M. (2020). Canagliflozin Prevents Diabetes-Induced Vascular Dysfunction in ApoE-Deficient Mice. J. Atheroscler. Thromb..

[104] Li F.F., Gao G., Li Q., Zhu H.H., Su X.F., Wu J.D., Ye L., Ma J.H. (2016). Influence of Dapagliflozin on Glycemic Variations in Patients with Newly Diagnosed Type 2 Diabetes Mellitus. J. Diabetes Res..

[105] Pignatelli P., Baratta F., Buzzetti R., D’Amico A., Castellani V., Bartimoccia S., Siena A., D’Onofrio L., Maddaloni E., Pingitore A. (2022). The Sodium-Glucose Co-Transporter-2 (SGLT2) Inhibitors Reduce Platelet Activation and Thrombus Formation by Lowering NOX2-Related Oxidative Stress: A Pilot Study. Antioxidants.

[106] Zhao S.M., Gao H.L., Wang Y.L., Xu Q., Guo C.Y. (2017). Attenuation of High Glucose-Induced Rat Cardiomyocyte Apoptosis by Exendin-4 via Intervention of HO-1/Nrf-2 and the PI3K/AKT Signaling Pathway. Chin. J. Physiol..

[107] Wu L., Wang K., Wang W., Wen Z., Wang P., Liu L., Wang D.W. (2018). Glucagon-like peptide-1 ameliorates cardiac lipotoxicity in diabetic cardiomyopathy via the PPARalpha pathway. Aging Cell..

[108] Nuamnaichati N., Mangmool S., Chattipakorn N., Parichatikanond W. (2020). Stimulation of GLP-1 Receptor Inhibits Methylglyoxal-Induced Mitochondrial Dysfunctions in H9c2 Cardiomyoblasts: Potential Role of Epac/PI3K/Akt Pathway. Front. Pharmacol..

[109] Qian P., Tian H., Wang Y., Lu W., Li Y., Ma T., Gao X., Yao W. (2020). A novel oral glucagon-like peptide 1 receptor agonist protects against diabetic cardiomyopathy via alleviating cardiac lipotoxicity induced mitochondria dysfunction. Biochem. Pharmacol..

[110] Zhang L., Tian J., Diao S., Zhang G., Xiao M., Chang D. (2020). GLP-1 receptor agonist liraglutide protects cardiomyocytes from IL-1beta-induced metabolic disturbance and mitochondrial dysfunction. Chem. Biol. Interact..

[111] Monji A., Mitsui T., Bando Y.K., Aoyama M., Shigeta T., Murohara T. (2013). Glucagon-like peptide-1 receptor activation reverses cardiac remodeling via normalizing cardiac steatosis and oxidative stress in type 2 diabetes. Am. J. Physiol. Heart Circ. Physiol..

[112] Ding W., Chang W.G., Guo X.C., Liu Y., Xiao D.D., Ding D., Wang J.X., Zhang X.J. (2019). Exenatide Protects Against Cardiac Dysfunction by Attenuating Oxidative Stress in the Diabetic Mouse Heart. Front. Endocrinol..

[113] Inoue T., Inoguchi T., Sonoda N., Hendarto H., Makimura H., Sasaki S., Yokomizo H., Fujimura Y., Miura D., Takayanagi R. (2015). GLP-1 analog liraglutide protects against cardiac steatosis, oxidative stress and apoptosis in streptozotocin-induced diabetic rats. Atherosclerosis.

[114] Li H., Xu X., Wang J., Kong X., Chen M., Jing T., Zhang Z., Yin G., Liu X., Hu Y. (2019). A Randomized Study to Compare the Effects of Once-Weekly Dulaglutide Injection and Once-Daily Glimepiride on Glucose Fluctuation of Type 2 Diabetes Mellitus Patients: A 26-Week Follow-Up. J. Diabetes Res..

[115] Zhang L., Qi X., Zhang G., Zhang Y., Tian J. (2020). Saxagliptin protects against hypoxia-induced damage in H9c2 cells. Chem. Biol. Interact..

[116] Lin C.P., Huang P.H., Chen C.Y., Wu M.Y., Chen J.S., Chen J.W., Lin S.J. (2021). Sitagliptin attenuates arterial calcification by downregulating oxidative stress-induced receptor for advanced glycation end products in LDLR knockout mice. Sci. Rep..

[117] Apaijai N., Pintana H., Chattipakorn S.C., Chattipakorn N. (2013). Effects of vildagliptin versus sitagliptin, on cardiac function, heart rate variability and mitochondrial function in obese insulin-resistant rats. Br. J. Pharmacol..

[118] Zhang X., Zhang Z., Yang Y., Suo Y., Liu R., Qiu J., Zhao Y., Jiang N., Liu C., Tse G. (2018). Alogliptin prevents diastolic dysfunction and preserves left ventricular mitochondrial function in diabetic rabbits. Cardiovasc. Diabetol..

[119] Zhang X., Zhang Z., Zhao Y., Jiang N., Qiu J., Yang Y., Li J., Liang X., Wang X., Tse G. (2017). Alogliptin, a Dipeptidyl Peptidase-4 Inhibitor, Alleviates Atrial Remodeling and Improves Mitochondrial Function and Biogenesis in Diabetic Rabbits. J. Am. Heart Assoc..

[120] Nath S., Ghosh S.K., Choudhury Y. (2017). A murine model of type 2 diabetes mellitus developed using a combination of high fat diet and multiple low doses of streptozotocin treatment mimics the metabolic characteristics of type 2 diabetes mellitus in humans. J. Pharmacol. Toxicol. Methods.

[121] Matsui T., Nishino Y., Takeuchi M., Yamagishi S. (2011). Vildagliptin blocks vascular injury in thoracic aorta of diabetic rats by suppressing advanced glycation end product-receptor axis. Pharmacol. Res..

[122] Aroor A.R., Sowers J.R., Bender S.B., Nistala R., Garro M., Mugerfeld I., Hayden M.R., Johnson M.S., Salam M., Whaley-Connell A. (2013). Dipeptidylpeptidase inhibition is associated with improvement in blood pressure and diastolic function in insulin-resistant male Zucker obese rats. Endocrinology.

[123] De Geest B., Mishra M. (2022). Role of Oxidative Stress in Diabetic Cardiomyopathy. Antioxidants.

[124] Fan D., Takawale A., Lee J., Kassiri Z. (2012). Cardiac fibroblasts, fibrosis and extracellular matrix remodeling in heart disease. Fibrogenesis Tissue Repair.

[125] Li J.H., Huang X.R., Zhu H.J., Oldfield M., Cooper M., Truong L.D., Johnson R.J., Lan H.Y. (2004). Advanced glycation end products activate Smad signaling via TGF-beta-dependent and independent mechanisms: Implications for diabetic renal and vascular disease. FASEB J..

[126] Peng Y., Kim J.M., Park H.S., Yang A., Islam C., Lakatta E.G., Lin L. (2016). AGE-RAGE signal generates a specific NF-kappaB RelA “barcode” that directs collagen I expression. Sci. Rep..

[127] Tuleta I., Frangogiannis N.G. (2021). Fibrosis of the diabetic heart: Clinical significance, molecular mechanisms, and therapeutic opportunities. Adv. Drug Deliv. Rev..

[128] Kong P., Christia P., Frangogiannis N.G. (2014). The pathogenesis of cardiac fibrosis. Cell. Mol. Life. Sci..

[129] Wong T.C., Piehler K.M., Kang I.A., Kadakkal A., Kellman P., Schwartzman D.S., Mulukutla S.R., Simon M.A., Shroff S.G., Kuller L.H. (2014). Myocardial extracellular volume fraction quantified by cardiovascular magnetic resonance is increased in diabetes and associated with mortality and incident heart failure admission. Eur. Heart J..

[130] Kang S., Verma S., Hassanabad A.F., Teng G., Belke D.D., Dundas J.A., Guzzardi D.G., Svystonyuk D.A., Pattar S.S., Park D.S.J. (2020). Direct Effects of Empagliflozin on Extracellular Matrix Remodelling in Human Cardiac Myofibroblasts: Novel Translational Clues to Explain EMPA-REG OUTCOME Results. Can. J. Cardiol..

[131] Osaka N., Mori Y., Terasaki M., Hiromura M., Saito T., Yashima H., Shiraga Y., Kawakami R., Ohara M., Fukui T. (2022). Luseogliflozin inhibits high glucose-induced TGF-beta2 expression in mouse cardiomyocytes by suppressing NHE-1 activity. J. Int. Med. Res..

[132] Verma S., Mazer C.D., Yan A.T., Mason T., Garg V., Teoh H., Zuo F., Quan A., Farkouh M.E., Fitchett D.H. (2019). Effect of Empagliflozin on Left Ventricular Mass in Patients With Type 2 Diabetes Mellitus and Coronary Artery Disease: The EMPA-HEART CardioLink-6 Randomized Clinical Trial. Circulation.

[133] Brown A.J.M., Gandy S., McCrimmon R., Houston J.G., Struthers A.D., Lang C.C. (2020). A randomized controlled trial of dapagliflozin on left ventricular hypertrophy in people with type two diabetes: The DAPA-LVH trial. Eur. Heart J..

[134] Wang X.W., Zhang F.X., Yang F., Ding Z.F., Agarwal N., Guo Z.K., Mehta J.L. (2016). Effects of linagliptin and liraglutide on glucose- and angiotensin II-induced collagen formation and cytoskeleton degradation in cardiac fibroblasts in vitro. Acta Pharmacol. Sin..

[135] Zhao T., Chen H., Cheng C., Zhang J., Yan Z., Kuang J., Kong F., Li C., Lu Q. (2019). Liraglutide protects high-glucose-stimulated fibroblasts by activating the CD36-JNK-AP1 pathway to downregulate P4HA1. Biomed. Pharmacother..

[136] Yu W., Zha W., Ren J. (2018). Exendin-4 and Liraglutide Attenuate Glucose Toxicity-Induced Cardiac Injury through mTOR/ULK1-Dependent Autophagy. Oxid. Med. Cell. Longev..

[137] Cai H., Zhou L., Liu J., Li Z., Chen S. (2022). Independent and combined effects of liraglutide and aerobic interval training on glycemic control and cardiac protection in diabetic cardiomyopathy rats. Biochem. Biophys. Res. Commun..

[138] Tate M., Robinson E., Green B.D., McDermott B.J., Grieve D.J. (2016). Exendin-4 attenuates adverse cardiac remodelling in streptozocin-induced diabetes via specific actions on infiltrating macrophages. Basic Res. Cardiol..

[139] Younce C.W., Niu J., Ayala J., Burmeister M.A., Smith L.H., Kolattukudy P., Ayala J.E. (2014). Exendin-4 improves cardiac function in mice overexpressing monocyte chemoattractant protein-1 in cardiomyocytes. J. Mol. Cell. Cardiol..

[140] Withaar C., Meems L.M.G., Markousis-Mavrogenis G., Boogerd C.J., Sillje H.H.W., Schouten E.M., Dokter M.M., Voors A.A., Westenbrink B.D., Lam C.S.P. (2021). The effects of liraglutide and dapagliflozin on cardiac function and structure in a multi-hit mouse model of heart failure with preserved ejection fraction. Cardiovasc. Res..

[141] Picatoste B., Ramirez E., Caro-Vadillo A., Iborra C., Ares-Carrasco S., Egido J., Tunon J., Lorenzo O. (2013). Sitagliptin reduces cardiac apoptosis, hypertrophy and fibrosis primarily by insulin-dependent mechanisms in experimental type-II diabetes. Potential roles of GLP-1 isoforms. PLoS ONE.

[142] Liu Y.S., Huang Z.W., Wang L., Liu X.X., Wang Y.M., Zhang Y., Zhang M. (2015). Sitagliptin alleviated myocardial remodeling of the left ventricle and improved cardiac diastolic dysfunction in diabetic rats. J. Pharmacol. Sci..

[143] Chen Y.F., Zhang L.J., Liu Q.H., Li X.W. (2021). Effects of Sitagliptin on myocardial remodeling and autophagy in diabetic mice and its mechanism. Chin. J. Appl. Physiol..

[144] Lenski M., Kazakov A., Marx N., Bohm M., Laufs U. (2011). Effects of DPP-4 inhibition on cardiac metabolism and function in mice. J. Mol. Cell. Cardiol..

[145] Moon J.Y., Woo J.S., Seo J.W., Lee A., Kim D.J., Kim Y.G., Kim S.Y., Lee K.H., Lim S.J., Cheng X.W. (2016). The Dose-Dependent Organ-Specific Effects of a Dipeptidyl Peptidase-4 Inhibitor on Cardiovascular Complications in a Model of Type 2 Diabetes. PLoS ONE.

[146] Bostick B., Habibi J., Ma L., Aroor A., Rehmer N., Hayden M.R., Sowers J.R. (2014). Dipeptidyl peptidase inhibition prevents diastolic dysfunction and reduces myocardial fibrosis in a mouse model of Western diet induced obesity. Metabolism.

[147] Hong S.K., Choo E.H., Ihm S.H., Chang K., Seung K.B. (2017). Dipeptidyl peptidase 4 inhibitor attenuates obesity-induced myocardial fibrosis by inhibiting transforming growth factor-betal and Smad2/3 pathways in high-fat diet-induced obesity rat model. Metabolism.

[148] Connelly K.A., Zhang Y., Advani A., Advani S.L., Thai K., Yuen D.A., Gilbert R.E. (2013). DPP-4 inhibition attenuates cardiac dysfunction and adverse remodeling following myocardial infarction in rats with experimental diabetes. Cardiovasc. Ther..

[149] Gu Y., Ma C.T., Gu H.L., Shi L., Tian X.T., Xu W.Q. (2018). Sitagliptin improves cardiac function after myocardial infarction through activation of autophagy in streptozotocin-induced diabetic mice. Eur. Rev. Med. Pharmacol. Sci..

[150] Nakajima Y., Ito S., Asakura M., Min K.D., Fu H.Y., Imazu M., Hitsumoto T., Takahama H., Shindo K., Fukuda H. (2019). A dipeptidyl peptidase-IV inhibitor improves diastolic dysfunction in Dahl salt-sensitive rats. J. Mol. Cell. Cardiol..

[151] Nikolaou P.E., Mylonas N., Makridakis M., Makrecka-Kuka M., Iliou A., Zerikiotis S., Efentakis P., Kampoukos S., Kostomitsopoulos N., Vilskersts R. (2022). Cardioprotection by selective SGLT-2 inhibitors in a non-diabetic mouse model of myocardial ischemia/reperfusion injury: A class or a drug effect?. Basic Res. Cardiol..

[152] Chan J.C.H., Chan M.C.Y. (2023). SGLT2 Inhibitors: The Next Blockbuster Multifaceted Drug?. Medicina.

